# Supplementary material for: Discovery of a maximally charged Weyl point
Source: Nat Commun. 2022 Nov 30;13:7359. doi: 10.1038/s41467-022-34978-z (PMC9712526; doi:10.1038/s41467-022-34978-z)
Supplement: Supplementary file 1 — Supplementary Information for Discovery of a maximally charged Weyl point [file 41467_2022_34978_MOESM1_ESM.pdf]

Supplementary Information

Discovery of a maximally charged Weyl point

Qiaolu Chen[1,2,3], Fujia Chen[1,2,3], Yuang Pan[1,2,3], Chaoxi Cui[4,5], Qinghui Yan[1,2,3], Li Zhang[1,2,3], Zhen Gao[6], Shengyuan A. Yang[7], Zhi-Ming Yu[4,5,*], Hongsheng Chen[1,2,3,*], Baile Zhang[8,9,*], Yihao Yang[1,2,3,*]

[1]Interdisciplinary Centre for Quantum Information, State Key Laboratory of Modern Optical Instrumentation, ZJU-Hangzhou Global Scientific and Technological Innovation Centre, Zhejiang University, Hangzhou 310027, China.

[2]International Joint Innovation Centre, Key Lab. of Advanced Micro/Nano Electronic Devices & Smart Systems of Zhejiang, The Electromagnetics Academy at Zhejiang University, Zhejiang University, Haining 314400, China.

[3]Jinhua Institute of Zhejiang University, Zhejiang University, Jinhua 321099, China.

[4]Centre for Quantum Physics, Key Laboratory of Advanced Optoelectronic Quantum Architecture and Measurement (MOE), School of Physics, Beijing Institute of Technology, Beijing 100081, China.

[5]Beijing Key Laboratory of Nanophotonics and Ultrafine Optoelectronic Systems, School of Physics, Beijing Institute of Technology, Beijing 100081, China.

[6]Department of Electrical and Electronic Engineering, Southern University of Science and Technology, Shenzhen 518055, China.

[7]Research Laboratory for Quantum Materials, Singapore University of Technology and Design, Singapore 487372, Singapore.

[8]Division of Physics and Applied Physics, School of Physical and Mathematical Sciences, Nanyang Technological University, 21 Nanyang Link, Singapore 637371, Singapore.

[9]Centre for Disruptive Photonic Technologies, The Photonics Institute, Nanyang Technological University, 50 Nanyang Avenue, Singapore 639798, Singapore.

*Correspondence to: (Y. Y.) yangyihao@zju.edu.cn; (Z. Y.) zhiming_yu@bit.edu.cn; (H. C.) hansomchen@zju.edu.cn; (B. Z.) blzhang@ntu.edu.sg.

Table of Contents

- **Supplementary Note 1.** Spin structures for the charge-1 Weyl point, charge-2 Weyl point, maximally charged Weyl point and charge-2 triple point.
- **Supplementary Note 2.** The helicoid, double-helicoid and quadruple-helicoid surface states in Fig. 1c.
- **Supplementary Note 3.** Detailed design of the 3D photonic crystal with maximally charged WP.
- **Supplementary Note 4.** Low-energy effective Hamiltonians for maximally charged WP, charge-2 triple point, and charge-2 WP.
- **Supplementary Note 5.** Numerical derivation of the matrix representations for maximally charged WP.
- **Supplementary Note 6.** Simulated and fitted cubic/quadratic dispersions around maximally charged WP.
- **Supplementary Note 7.** First-principle calculations of the topological charges with Wilson loop method.
- **Supplementary Note 8.** Numerical evidence for the topological charges of the maximally charged WP and the charge-2 WP.
- **Supplementary Note 9.** Numerical evidence for the topological charge of the charge-2 triple point.
- **Supplementary Note 10.** The evolution of maximally charged WP under $C_{3,111}$ symmetry breaking.
- **Supplementary Note 11.** Design of a 3D acoustic crystal with a maximally charged WP.
- **Supplementary Note 12.** The projections of WPs on the lowest three bands on k_x-k_y plane, and the surface dispersion.
- **Supplementary Fig. 1 |** Tight-binding lattice model hosting the maximally charged WP.
- **Supplementary Fig. 2 |** Simulated electric and magnetic fields of two degenerate modes at the maximally charged WP.

- **Supplementary Fig. 3 |** Numerically simulated (red circles) and fitted (black curves) band structures in the vicinity of Γ point along <111> and <101> directions, respectively.
- **Supplementary Fig. 4 |** Evolution of the Wannier centres on the spheres enclosing maximally charged WP, charge-2 triple point, charge-2 WP and charge-1 WP.
- **Supplementary Fig. 5 |** Numerical evidence for the topological charges of the maximally charged WP and the charge-2 WP.
- **Supplementary Fig. 6 |** Numerical evidence for the topological charge of the charge-2 triple point.
- **Supplementary Fig. 7 |** The evolution of maximally charged WP under $C_{3,111}$ symmetry breaking.
- **Supplementary Fig. 8 |** The topological surface states and Fermi arcs on k_x-k_y plane when $C_{3,111}$ symmetry is broken.
- **Supplementary Fig. 9 |** The topological surface states and Fermi arcs on k_y-k_z plane when $C_{3,111}$ symmetry is broken.
- **Supplementary Fig. 10 |** Design of a 3D acoustic crystal with a maximally charged WP.
- **Supplementary Fig. 11 |** Projected WPs of the lowest three bands on k_x-k_y plane, and the corresponding surface states.
- **Supplementary Fig. 12 |** Detailed schematic of the unit cell of the self-supporting 3D photonic crystal.
- **Supplementary Fig. 13 |** Measured surface iso-frequency contours at 4.7, 4.8 and 5.5 GHz.
- **Supplementary Fig. 14 |** Experimental setup consisting of a vector network analyser (VNA), two cables, a source, a probe, and a fabricated sample.

Supplementary Note 1. Spin structures for the charge-1 Weyl point, charge-2 Weyl point, maximally charged Weyl point and charge-2 triple point.

Generally, a doubly degenerate Weyl point (WP) with topological charge C ($|C|$ = 1, 2, 3) has the low-energy effective **k·p** model that can be written as[1],

$$H_N(\mathbf{k}) = \begin{bmatrix} Ak_z & B(k_x - ik_y)^N \\ B(k_x + ik_y)^N & -Ak_z \end{bmatrix} \tag{1}$$

where A and B are real constants. Besides, the low-energy effective Hamiltonian of a maximally charged WP with $C_{3,111}$ rotation symmetry along [111] direction can be obtained as[2],

$$H_4(\mathbf{k}) = \begin{bmatrix} Ak_xk_yk_z & B\left[k^2 - 3k_z^2 - i\sqrt{3}\left(k_x^2 - k_y^2\right)\right] \\ B\left[k^2 - 3k_z^2 + i\sqrt{3}\left(k_x^2 - k_y^2\right)\right] & -Ak_xk_yk_z \end{bmatrix}, \tag{2}$$

where $k = \sqrt{k_x^2 + k_y^2 + k_z^2}$.

These two two-fold models can also be denoted as $H_{2\times2}(\mathbf{k}) = \sum_{i=x,y,z} f_i\,(\mathbf{k}) \cdot \sigma_i$, where $f_i(\mathbf{k})$ represent the spin texture and σ_i is the Pauli matrix. Here, the spin is defined as[1],

$$\hat{S}_i \equiv \frac{f_i(\mathbf{k})}{|\mathbf{f}(\mathbf{k})|} = \langle \psi_{up} | \sigma_i | \psi_{up} \rangle \tag{3}$$

where ψ_{up} is the wave function of the upper band in the vicinity of the WP.

Based on the above Hamiltonian, we can obtain the spin structures for charge-1 WP, charge-2 WP and maximally charged WP, which appear in the photonic crystal of this work (see Fig. 1b in the main text). For simplicity but without loss of generality, we suppose the real constants $A = B$. For charge-1 WP ($|C| = 1$), the spin can be written as,

$$\hat{S}_i = \frac{k_i}{|\mathbf{k}|}, i = x, y, z. \tag{4}$$

For charge-2 WP ($|C| = 2$), the spin is,

$$\hat{S}_x = \frac{k_x^2 - k_y^2}{\sqrt{(k_x^2 + k_y^2)^2 + k_z^2}}, \tag{5}$$

$$\hat{S}_y = \frac{2k_xk_y}{\sqrt{(k_x^2 + k_y^2)^2 + k_z^2}}, \tag{6}$$

$$\hat{S}_z = \frac{k_z}{\sqrt{(k_x^2 + k_y^2)^2 + k_z^2}}. \tag{7}$$

As for maximally charged WP, the spin is,

$$\hat{S}_x = \frac{k_x^2 + k_y^2 - 2k_z^2}{|\mathbf{f}(\mathbf{k})|}, \tag{8}$$

$$\hat{S}_y = \frac{\sqrt{3}\left(k_x^2 - k_y^2\right)}{|\mathbf{f}(\mathbf{k})|}, \tag{9}$$

$$\hat{S}_z = \frac{k_x k_y k_z}{|\mathbf{f}(\mathbf{k})|}, \tag{10}$$

with $|\mathbf{f}(\mathbf{k})| = \sqrt{2\left[(k_x^2 - k_y^2)^2 + (k_y^2 - k_z^2)^2 + (k_z^2 - k_x^2)^2\right] + k_x^2 k_y^2 k_z^2}$.

The band structure of a charge-2 triple point can be described by a three-band effective Hamiltonian $H_{TP}(\mathbf{k}) = \mathbf{k} \cdot \mathbf{L}$, with $\mathbf{L}$ being the spin-1 matrix representation[3-5]:

$$L_x = \begin{bmatrix} 0 & 0 & 0 \\ 0 & 0 & -i \\ 0 & i & 0 \end{bmatrix}, \tag{11}$$

$$L_y = \begin{bmatrix} 0 & 0 & -i \\ 0 & 0 & 0 \\ i & 0 & 0 \end{bmatrix}, \tag{12}$$

$$L_z = \begin{bmatrix} 0 & -i & 0 \\ i & 0 & 0 \\ 0 & 0 & 0 \end{bmatrix}. \tag{13}$$

Similarly, the spin for charge-2 triple point is defined as,

$$\hat{S}_i \equiv \frac{f_i(\mathbf{k})}{|\mathbf{f}(\mathbf{k})|} = \langle \psi_{up} | L_i | \psi_{up} \rangle \tag{14}$$

Straightforward calculations give

$$\hat{S}_i = \frac{k_i}{|\mathbf{k}|}, i = x, y, z, \tag{15}$$

which is identical to that of charge-1 WP.

Supplementary Note 2. The helicoid, double-helicoid and quadruple-helicoid surface states in Fig. 1c.

In surface momentum space, the surface state of the charge-1 WP is topologically equivalent to a helicoidal structure, corresponding to a non-compact Riemann surface described by the generating function of $\omega(k_x, k_y) \sim \mathrm{Im}[\log(k_x + ik_y)]$[5-6]. For the WP with a larger Chern number ($|C| > 1$), the surface state contains $|C|$ sheet and each sheet can be considered as a helicoidal structure with the same chirality. Thus, the surface state of charge-2 (maximally charged) WP is topologically equivalent to the superposition of 2 (4) helicoids, leading to a double-helicoid (quadruple-helicoid) surface state. The double-helicoid (quadruple-helicoid) surface state is topologically equivalent to non-compact Riemann surfaces of $\omega(k_x, k_y) \sim \mathrm{Im}[\log \sqrt[n]{(k_x + ik_y)^n}]$ with $n = 2$ ($n = 4$)[5-6].

Supplementary Note 3. Detailed design of the 3D photonic crystal with maximally charged WP.

It has been proposed that the maximally charged WP could be realised in systems without spin-orbit coupling, with space groups (SGs) No. 195-199 and 207-214[7]. The point group for SGs No. 195-199 is *T,* and for No. 207-214 is *O*. Among the 14 SG candidates, SG No. 207 may be the most convenient one for experimental design, as it is a symmorphic SG with a simple cubic Bravais lattice and hosts higher point group symmetry (compared with *T* point group). Hence, we decide to design a crystal belonging to SG No. 207 to realise the maximally charged WP.

We start with a tight-binding lattice model. After iterating through various Wyckoff positions, we obtain a tight-binding lattice model, which possesses the maximally charged WP with clear quadruple-helicoid surface arc states over a large energy window, which are important for experimental implementation. The lattice, as shown in Supplementary Fig. 1a, has six sites per unit cell $\{A_{i=1,6}\}$ locating at $6e$ Wyckoff positions of SG No. 207: $\{x, 0, 0\}$, $\{0, x, 0\}$, $\{0, 0, x\}$, $\{-x, 0, 0\}$, $\{0, -x, 0\}$ and $\{0, 0, -x\}$, respectively. The three lattice vectors for this lattice are $\{a, 0, 0\}$, $\{0, a,$

0} and {0, 0, a}, with a being the lattice constant. We here set $x = a/5$, and each site has a s-like orbital without a spin degree of freedom. The symmetry operators of SG No. 207 are symmorphic and can be generated by $C_{3,111}$, C_{2z}, C_{2x}, $C_{2,110}$. This lattice model also has time-reversal symmetry. With the symmetry constraints, the lattice Hamiltonian can be written as,

$$H = t_1 \begin{bmatrix} 0 & e^{i\left(\frac{k_y}{5}-\frac{k_x}{5}\right)} & e^{i\left(\frac{k_z}{5}-\frac{k_x}{5}\right)} & 0 & e^{i\left(-\frac{k_x}{5}-\frac{k_y}{5}\right)} & e^{i\left(-\frac{k_x}{5}-\frac{k_z}{5}\right)} \\ e^{i\left(\frac{k_x}{5}-\frac{k_y}{5}\right)} & 0 & e^{i\left(\frac{k_z}{5}-\frac{k_y}{5}\right)} & e^{i\left(-\frac{k_x}{5}-\frac{k_y}{5}\right)} & 0 & e^{i\left(-\frac{k_z}{5}-\frac{k_y}{5}\right)} \\ e^{i\left(\frac{k_x}{5}-\frac{k_z}{5}\right)} & e^{i\left(\frac{k_y}{5}-\frac{k_z}{5}\right)} & 0 & e^{i\left(-\frac{k_x}{5}-\frac{k_z}{5}\right)} & e^{i\left(-\frac{k_y}{5}-\frac{k_z}{5}\right)} & 0 \\ 0 & e^{i\left(\frac{k_x}{5}+\frac{k_y}{5}\right)} & e^{i\left(\frac{k_x}{5}+\frac{k_z}{5}\right)} & 0 & e^{i\left(\frac{k_x}{5}-\frac{k_y}{5}\right)} & e^{i\left(\frac{k_x}{5}-\frac{k_z}{5}\right)} \\ e^{i\left(\frac{k_x}{5}+\frac{k_y}{5}\right)} & 0 & e^{i\left(\frac{k_y}{5}+\frac{k_z}{5}\right)} & e^{i\left(\frac{k_y}{5}-\frac{k_x}{5}\right)} & 0 & e^{i\left(\frac{k_y}{5}-\frac{k_z}{5}\right)} \\ e^{i\left(\frac{k_x}{5}+\frac{k_z}{5}\right)} & e^{i\left(\frac{k_y}{5}+\frac{k_z}{5}\right)} & 0 & e^{i\left(\frac{k_z}{5}-\frac{k_x}{5}\right)} & e^{i\left(\frac{k_z}{5}-\frac{k_y}{5}\right)} & 0 \end{bmatrix}$$

$$+t_2 \begin{bmatrix} 0 & 0 & 0 & e^{\frac{3ik_x}{5}} & 0 & 0 \\ 0 & 0 & 0 & 0 & e^{\frac{3ik_y}{5}} & 0 \\ 0 & 0 & 0 & 0 & 0 & e^{\frac{3ik_z}{5}} \\ e^{-\frac{3ik_x}{5}} & 0 & 0 & 0 & 0 & 0 \\ 0 & e^{-\frac{3ik_y}{5}} & 0 & 0 & 0 & 0 \\ 0 & 0 & e^{-\frac{3ik_z}{5}} & 0 & 0 & 0 \end{bmatrix}$$

$$+t_3 \begin{bmatrix} 0 & e^{i\left(-\frac{k_x}{5}+\frac{k_y}{5}+k_z\right)} & e^{i\left(-\frac{k_x}{5}-k_y+\frac{k_z}{5}\right)} & 0 & e^{i\left(-\frac{k_x}{5}-\frac{k_y}{5}-k_z\right)} & e^{i\left(-\frac{k_x}{5}+k_y-\frac{k_z}{5}\right)} \\ e^{i\left(\frac{k_x}{5}-\frac{k_y}{5}-k_z\right)} & 0 & e^{i\left(k_x-\frac{k_y}{5}+\frac{k_z}{5}\right)} & e^{i\left(-\frac{k_x}{5}-\frac{k_y}{5}+k_z\right)} & 0 & e^{i\left(-k_x-\frac{k_y}{5}-\frac{k_z}{5}\right)} \\ e^{i\left(\frac{k_x}{5}+k_y-\frac{k_z}{5}\right)} & e^{i\left(-k_x+\frac{k_y}{5}-\frac{k_z}{5}\right)} & 0 & e^{i\left(-\frac{k_x}{5}-k_y-\frac{k_z}{5}\right)} & e^{i\left(k_x-\frac{k_y}{5}-\frac{k_z}{5}\right)} & 0 \\ 0 & e^{i\left(\frac{k_x}{5}+\frac{k_y}{5}-k_z\right)} & e^{i\left(\frac{k_x}{5}+k_y+\frac{k_z}{5}\right)} & 0 & e^{i\left(\frac{k_x}{5}-\frac{k_y}{5}+k_z\right)} & e^{i\left(\frac{k_x}{5}-k_y-\frac{k_z}{5}\right)} \\ e^{i\left(\frac{k_x}{5}+\frac{k_y}{5}+k_z\right)} & 0 & e^{i\left(-k_x+\frac{k_y}{5}+\frac{k_z}{5}\right)} & e^{i\left(-\frac{k_x}{5}+\frac{k_y}{5}-k_z\right)} & 0 & e^{i\left(k_x+\frac{k_y}{5}-\frac{k_z}{5}\right)} \\ e^{i\left(\frac{k_x}{5}-k_y+\frac{k_z}{5}\right)} & e^{i\left(k_x+\frac{k_y}{5}+\frac{k_z}{5}\right)} & 0 & e^{i\left(-\frac{k_x}{5}+k_y+\frac{k_z}{5}\right)} & e^{i\left(-k_x-\frac{k_y}{5}+\frac{k_z}{5}\right)} & 0 \end{bmatrix}. \tag{16}$$

The hopping is $t_1 = 0.06$, $t_2 = 1$, and $t_3 = 0.04$. The hopping graphs for t_1 and t_2 are shown in Supplementary Fig. 1b, while that for t_3 is very complicated. Instead, we can understand the hopping t_3 as following relations:

$$A_1 \rightarrow [0, A_2 + (001), A_3 - (010), 0, A_5 - (001), A_6 + (010)]$$

$$A_2 \rightarrow [A_1 - (001), 0, A_3 + (100), A_4 + (001), 0, A_6 - (100)]$$

$$A_3 \rightarrow [A_1 + (010), A_2 - (100), 0, A_4 - (010), A_5 + (100), 0]$$

$$A_4 \rightarrow [0, A_2 - (001), A_3 + (010), 0, A_5 + (001), A_6 - (010)]$$

$$A_5 \rightarrow [A_1 + (001), 0, A_3 - (100), A_4 - (001), 0, A_6 + (100)]$$

$$A_6 \to [A_1 - (010), A_2 + (100), 0, A_4 + (010), A_5 - (100), 0]. \tag{17}$$

The bulk band structure of the tight-binding Hamiltonian is shown in Supplementary Fig. 1c. One can find that there exist various band crossings, such as a maximally charged WP (red dot) with topological charge $|C| = 4$ at R, a charge-2 triple point (blue dot) with topological charge $|C| = 2$ at Γ, and six conventional WPs (green dots) with topological charge $|C| = 1$ at Γ-X, Γ-Y and Γ-Z paths. By calculating the projected surface spectrum on the (100) surface (see Supplementary Fig. 1e) and the constant energy ($E_F = -1.04$) slice (see Supplementary Fig. 1f), one can observe that four Fermi arcs emanate from the projection of the maximally charged WP at $\overline{\mathrm{M}}$.

However, the complicated hopping $t_{i=1,2,3}$ is difficult to achieve in realistic 3D photonic crystals. Thus, we continue to refine our results based on COMSOL Multiphysics software. As the maximally charged WP is symmetry enforced, we design a 3D photonic crystal by maintaining the symmetry. We find that by moving "atoms" to 3*d* Wyckoff position, replacing t_3 bonds with perfect electric conductor (PEC) rods and removing all t_1 and t_2 bonds, the resulting structure is elegant, and the space group remains No. 207.

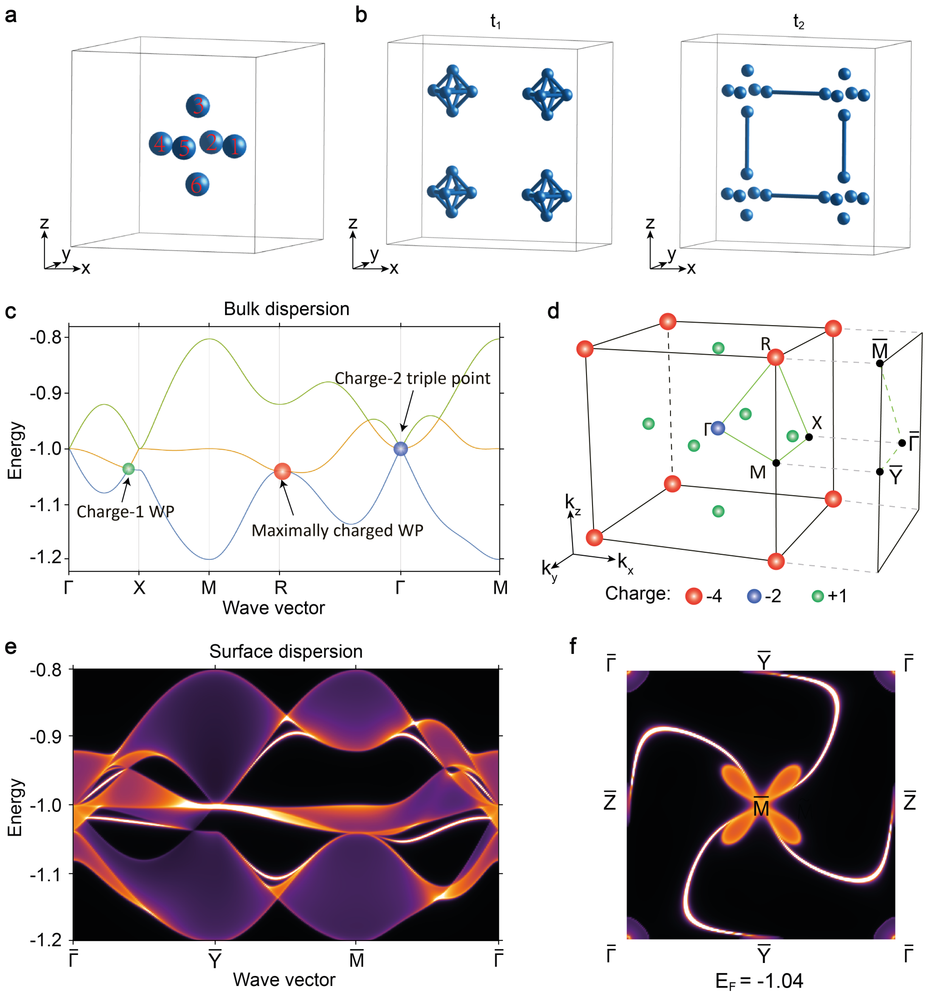

Supplementary Fig. 1 | Tight-binding lattice model hosting the maximally charged WP. a Schematic view of the unit cell, with six sites $\{A_{i=1,6}\}$ locating at $\{x, 0, 0\}$, $\{0, x, 0\}$, $\{0, 0, x\}$, $\{-x, 0, 0\}$, $\{0,-x, 0\}$ and $\{0, 0,-x\}$, and $x = a/5$. a is the lattice constant. **b** Schematics of hopping t_1 and t_2 in four unit cells. **c**,**d** Band structure (c) and 3D first Brillouin zone (d) for the tight-binding lattice model with a maximally charged WP (red dot), a charge-2 triple point (blue dot) and six charge-1 WPs (green dots). **e** Projected surface spectrum on the (100) surface for the tight-binding lattice model. **f** The slice at constant energy ($E_F = -1.04$) around the maximally charged WP energy. Four Fermi arcs emerge from the projection of the maximally charged WP at $\overline{\mathrm{M}}$.

Supplementary Note 4. Low-energy effective Hamiltonians for maximally charged WP, charge-2 triple point, and charge-2 WP.

To characterise the band degenerate points in the photonic crystal, we construct the low-energy effective Hamiltonians around the maximally charged WP at Γ point, the charge-2 triple point at R point, and the charge-2 WP at M point, respectively. At Γ point, the generating elements of the corresponding little group are $C_{3,111}$, C_{2z}, C_{2x}, $C_{2,110}$,

and τ. The matrix representation of the generating elements for the maximally charged WP can be written as

$$C_{3,111} = \frac{1}{2}\begin{bmatrix} -1 & \sqrt{3} \\ -\sqrt{3} & -1 \end{bmatrix}, \tag{18}$$

$$C_{2z} = \begin{bmatrix} 1 & 0 \\ 0 & 1 \end{bmatrix}, \tag{19}$$

$$C_{2x} = \begin{bmatrix} 1 & 0 \\ 0 & 1 \end{bmatrix}, \tag{20}$$

$$C_{2,110} = \begin{bmatrix} 1 & 0 \\ 0 & -1 \end{bmatrix}, \tag{21}$$

and

$$\tau = \begin{bmatrix} 1 & 0 \\ 0 & 1 \end{bmatrix} K, \tag{22}$$

with K being a complex conjugation operator. Then the effective Hamiltonian of the maximally charged WP should be invariant under the above symmetries, and can be obtained as (up to leading order)

$$H_\Gamma = c_1 + c_2 k^2 + \begin{bmatrix} \frac{k_x^2 + k_y^2 - 2k_z^2}{\sqrt{3}} c_3 & c_3\left(k_x^2 - k_y^2\right) + i c_4 k_x k_y k_z \\ c_3\left(k_x^2 - k_y^2\right) - i c_4 k_x k_y k_z & -\frac{k_x^2 + k_y^2 - 2k_z^2}{\sqrt{3}} c_3 \end{bmatrix}, \tag{23}$$

where c_i ($i = 1, 2, 3, 4$) are real parameters, and $k = \sqrt{k_x^2 + k_y^2 + k_z^2}$. This Hamiltonian H_Γ is connected to $H_4(\mathbf{k})$ in Supplementary Note 1 by a unitary transformation.

R point shares the same symmetry condition as Γ point, and the matrix representation of the generating elements for the charge-2 triple point can be written as

$$C_{3,111} = \begin{bmatrix} 0 & 1 & 0 \\ 0 & 0 & 1 \\ 1 & 0 & 0 \end{bmatrix}, \tag{24}$$

$$C_{2z} = \begin{bmatrix} -1 & 0 & 0 \\ 0 & -1 & 0 \\ 0 & 0 & 1 \end{bmatrix}, \tag{25}$$

$$C_{2x} = \begin{bmatrix} 1 & 0 & 0 \\ 0 & 1 & 0 \\ 0 & 0 & 1 \end{bmatrix}, \tag{26}$$

$$C_{2,110} = \begin{bmatrix} 0 & 1 & 0 \\ 1 & 0 & 0 \\ 0 & 0 & -1 \end{bmatrix}, \tag{27}$$

and

$$\tau = \begin{bmatrix} e^{-i\frac{2\pi}{3}} & 0 & 0 \\ 0 & e^{-i\frac{2\pi}{3}} & 0 \\ 0 & 0 & e^{-i\frac{2\pi}{3}} \end{bmatrix} K. \tag{28}$$

The effective Hamiltonian of the charge-2 triple point then can be obtained as (up to leading order)

$$H_{\mathrm{R}} = c_5 + c_6 \mathbf{k} \cdot \mathbf{L} \tag{29}$$

where c_i (i = 5, 6) are real parameters, and $\mathbf{L} = \{L_x, L_y, L_z\}$ is the spin-1 matrix representation satisfying the algebra of angular momentum.

The charge-2 WP at M point is protected by C_{4z}, C_{2x}, and τ symmetries, for which the matrix representation can be written as

$$C_{4z} = \begin{bmatrix} 0 & -i \\ i & 0 \end{bmatrix}, \tag{30}$$

$$C_{2x} = \begin{bmatrix} 1 & 0 \\ 0 & -1 \end{bmatrix}, \tag{31}$$

and

$$\tau = \begin{bmatrix} 1 & 0 \\ 0 & 1 \end{bmatrix} K. \tag{32}$$

Then the effective Hamiltonian of the charge-2 WP can be obtained as (up to leading order)

$$H_{\mathrm{M}} = c_7 + c_8\left(k_x^2 + k_y^2\right) + c_9 k_z^2 + \begin{bmatrix} c_{10}\left(k_x^2 - k_y^2\right) & c_{11}k_x k_y + i c_{12} k_z \\ c_{11} k_x k_y - i c_{12} k_z & -c_{10}\left(k_x^2 - k_y^2\right) \end{bmatrix}, \tag{33}$$

where c_i (i = 7, 8, 9, 10, 11, 12) are real parameters.

Supplementary Note 5. Numerical derivation of the matrix representations for maximally charged WP.

In this section, we give the details for mode analysis and numerical calculation of the matrix representations of the generators of the little group at the maximally charged WP.

We calculate the matrix representations by utilizing first-principles simulated electric fields of two degenerate eigenmodes at the maximally charged WP in the commercial software COMSOL, i.e., $\mathbf{E}_{01}(\mathbf{r})$ and $\mathbf{E}_{02}(\mathbf{r})$ (see Supplementary Fig. 2). The normalized electric fields of the modes are,

$$\mathbf{E}_i(\mathbf{r}) = \frac{\mathbf{E}_{0i}(\mathbf{r})}{\left(\int d\mathbf{r}\, \mathbf{E}_{0i}^*(\mathbf{r})\mathbf{E}_{0i}(\mathbf{r})d\mathbf{r}\right)^{-\frac{1}{2}}} \quad (i = 1,2). \tag{34}$$

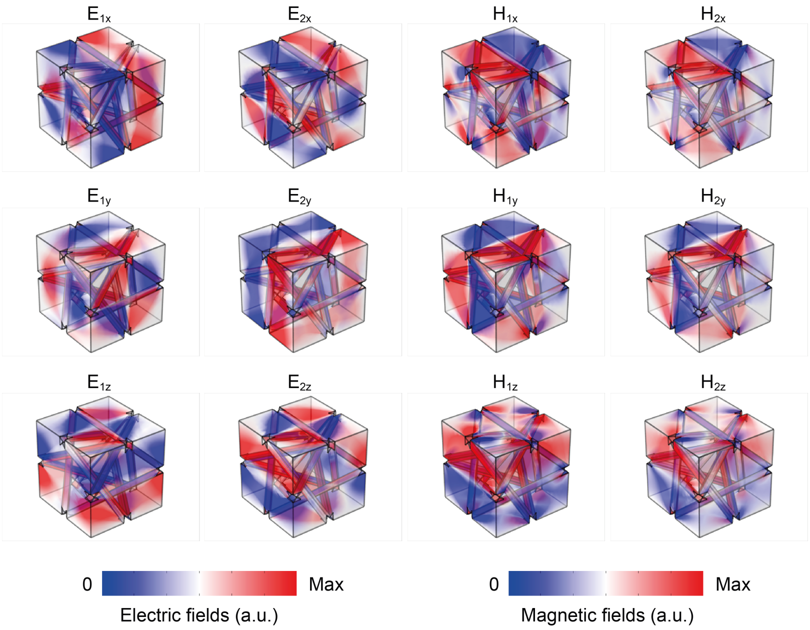

Supplementary Fig. 2 | Simulated electric and magnetic fields of two degenerate modes at the maximally charged WP.

Since the two modes are degenerate, an arbitrary linear combination of these two modes is also one of the eigenmodes of the degenerate point. In order to obtain the same matrix representations as Eq. 18-21, we apply a unitary transformation on $\mathbf{E}_i(\mathbf{r})$ to $\mathbf{E}_i'(\mathbf{r})$. The unitary transformation could be written as,

$$\begin{bmatrix} \mathbf{E}_1'(\mathbf{r}) \\ \mathbf{E}_2'(\mathbf{r}) \end{bmatrix} = U \begin{bmatrix} \mathbf{E}_1(\mathbf{r}) \\ \mathbf{E}_2(\mathbf{r}) \end{bmatrix}. \tag{35}$$

Based on $\mathbf{E}_i'(\mathbf{r})$, the matrix representations $D(R)$ corresponding to the symmetric operation R can be derived as,

$$D(R) = \begin{bmatrix} \int \mathbf{E}_1'(\mathbf{r}) R\mathbf{E}_1'(R^{-1}\mathbf{r})\, d\mathbf{r} & \int \mathbf{E}_1'(\mathbf{r}) R\mathbf{E}_2'(R^{-1}\mathbf{r})\, d\mathbf{r} \\ \int \mathbf{E}_2'(\mathbf{r}) R\mathbf{E}_1'(R^{-1}\mathbf{r})\, d\mathbf{r} & \int \mathbf{E}_2'(\mathbf{r}) R\mathbf{E}_2'(R^{-1}\mathbf{r})\, d\mathbf{r} \end{bmatrix} \tag{36}$$

where $R\mathbf{E}_i'$ represents the operation that applies symmetric operation R to $\mathbf{E}_i'$, and $R^{-1}\mathbf{r}$ is to apply inversed symmetric operation R^{-1} to $\mathbf{r}$. Thus, we numerically obtain the four matrix representations for the maximally charged WP,

$$C_{3,111} \approx \frac{1}{2}\begin{bmatrix} -1 & \sqrt{3} \\ -\sqrt{3} & -1 \end{bmatrix}, \tag{37}$$

$$C_{2z} \approx \begin{bmatrix} 1 & 0 \\ 0 & 1 \end{bmatrix}, \tag{38}$$

$$C_{2x} \approx \begin{bmatrix} 1 & 0 \\ 0 & 1 \end{bmatrix}, \tag{39}$$

$$C_{2,110} \approx \begin{bmatrix} 1 & 0 \\ 0 & -1 \end{bmatrix}. \tag{40}$$

One can observe that, the matrix representations are consistent with the theoretical matrix representations Eq. 18-21 in Supplementary Note 4.

In summary, based on the numerically simulated electric fields of the modes at the maximally charged WP, we calculate the corresponding matrix representations of the generators of the little group, which are the same as the theoretical analysis (i.e., Eq. 18-21 in Supplementary Note 4). We thus can directly obtain the low-energy effective Hamiltonian (see the details in Supplementary Note 4). We note that the matrix representations for the charge-2 WP and charge-2 triple point can also be derived in the same way.

Supplementary Note 6. Simulated and fitted cubic/quadratic dispersions around maximally charged WP.

Based on the effective Hamiltonian of the maximally charged WP, the dispersion relations along [111] and [101] directions can be written as,

$$\omega_{111} = c_1 + 3c_2k_x^2 \pm c_4k_x^3 \tag{41}$$

$$\omega_{101} = c_1 + \left(2c_2 \pm \frac{2\sqrt{3}}{3}c_3\right)k_x^2. \tag{42}$$

We then numerically fit the simulated dispersions with Eq. 41 and Eq. 42, with $c_1 = 3.5 \times 10^{10}$, $c_2 = -1.4 \times 10^6$, $c_3 = 9.2 \times 10^5$, $c_4 = 4.0 \times 10^4$, as shown in Supplementary Fig. 3, proving the cubic dispersions along [111] and quadratic dispersions along [101].

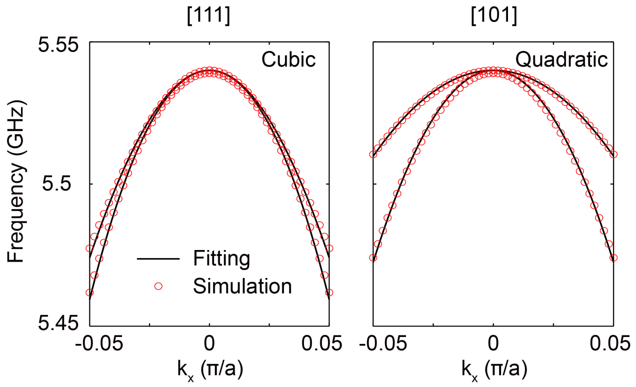

Supplementary Fig. 3 | Numerically simulated (red circles) and fitted (black curves) band structures in the vicinity of Γ point along [111] and [101] directions, respectively. The plotted range is $[-0.05\pi/a, 0.05/a]$.

Supplementary Note 7. First-principle calculations of the topological charges with Wilson loop method.

We calculate the topological charges for the maximally charged WP, the charge-2 triple point, the charge-2 WP, and the charge-1 WPs, respectively. We first track the evolution of Wannier centres on a sphere covering four kinds of band degenerate points. The sphere is discretized into a sequence of horizontal loops, from the north pole to the south pole of the sphere (i.e., the polar angle θ in spherical coordinates varies from 0 to π). Then the Berry phase along these horizontal loops can be numerically calculated by employing the Wilson loop method[5,8-9], in which the wavefunctions are extracted from the COMSOL Multiphysics calculations. The Wannier centres (ϕ) are simply the trace of the Berry phase.

Supplementary Fig. 4 shows the Wannier centres for the lower band of the maximally charged WP, the charge-2 triple point, the charge-2 WP, and the charge-1 WPs. For the maximally charged WP at Γ point (Supplementary Fig. 4a), the calculated Wannier centres shift by −8π, revealing that the topological charge of the lower band is −4. Similarly, the topological charges of the lower band for the charge-2 triple point and the charge-2 WP are −2 and +2, respectively (Supplementary Figs. 4b and 4c). Besides, the charge-1 WPs between M and R, and between R and Γ have the same topological charge of +1, while that between R and X has topological charge of −1, as shown in Supplementary Figs. 4d to 4f.

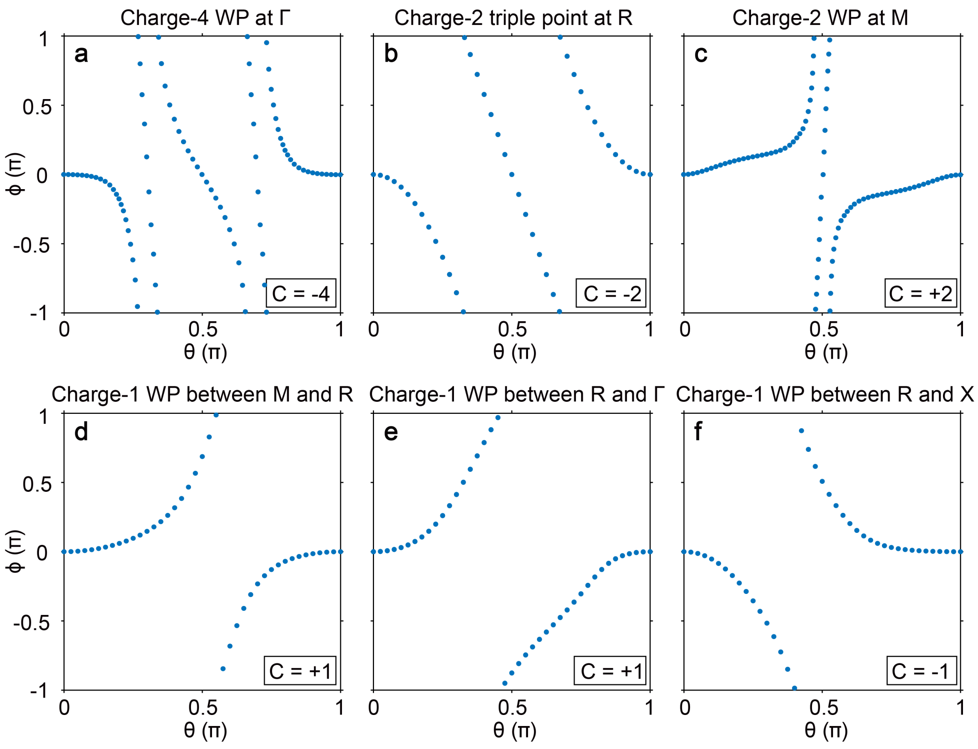

Supplementary Fig. 4 | Evolution of the Wannier centres on the spheres enclosing maximally charged WP, charge-2 triple point, charge-2 WP and charge-1 WP. a-f Wannier centres for the lower band of the maximally charged WP at Γ (**a**), the charge-2 triple point at R (**b**), the charge-2 WP at M (**c**), and the charge-1 WPs at general momenta (**d-f**), respectively.

Supplementary Note 8. Numerical evidence for the topological charges of the maximally charged WP and the charge-2 WP.

We can also identify the topological charges of the WPs by checking the chirality of the topological surface states along the closed loops, which encircle the projected $\bar{\Gamma}$ and $\bar{X}$ points, respectively[10-12]. As schematically shown in Supplementary Fig. 5a, a tube orientated along the k_z direction in the centre of the 3D Brillouin zone is projected onto the surface Brillouin zone, forming a closed loop around the $\bar{\Gamma}$ point. Supplementary Fig. 5b shows the projected surface dispersions along the clockwise circular paths around the $\bar{\Gamma}$ and $\bar{X}$ points, with radii 0.64 π/a and 0.27 π/a, respectively. Obviously, four (two) gapless topological surface states with overall negative (positive) slopes are observed inside the full bandgap, indicating the topological charge −4 (+2) of the WP at $\bar{\Gamma}$ ($\bar{X}$) point.

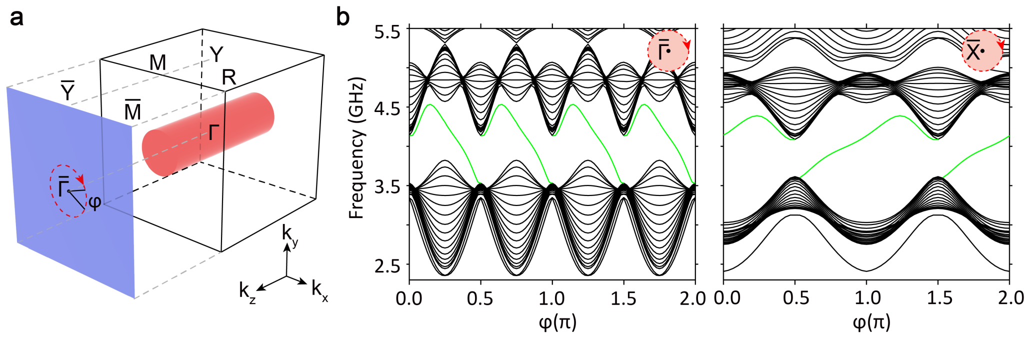

Supplementary Fig. 5 | Numerical evidence for the topological charges of the maximally charged WP and the charge-2 WP. a The 3D Brillouin zone and its surface projection on the k_x-k_y plane. The red dashed circle encircling the $\bar{\Gamma}$ point is the projection of the red tube oriented along k_z direction. **b** The surface dispersions along the clockwise circular paths around the $\bar{\Gamma}$ and $\bar{\mathrm{X}}$ points, with radii of 0.64 π/a and 0.27 π/a, respectively. The green curves represent the topological surface states.

Supplementary Note 9. Numerical evidence for the topological charge of the charge-2 triple point.

Since the topological charge of the charge-2 triple point ($C = -2$) is offset by the charge-2 WP ($C = +2$) when projecting the 3D band structure onto the k_x-k_y plane, the topological charge of the triple point could not be directly identified. In this section, we consider a super cell of the 3D metallic-mesh photonic crystal (see Supplementary Fig. 6a), with lattice constant of a = 15 mm along [010] direction, and $\sqrt{2}a$ along [100] and [001] directions. The corresponding 3D Brillouin zone is shown in Supplementary Fig. 6b (grey cuboid), whose volume is reduced by a factor of 2. It is obvious that the charge-2 triple point now resides at Y’. We then examine the surface dispersion along a circular loop enclosing the projection of the charge-2 triple point at $\bar{\mathrm{Y}}'$, as schematically shown in Supplementary Fig. 6c. This loop has a radius of 0.23 π/a. The surface dispersion plotted in Supplementary Fig. 6d indicates two gapless topological surface states with overall negative slopes, revealing the topological charge of the triple point is indeed −2.

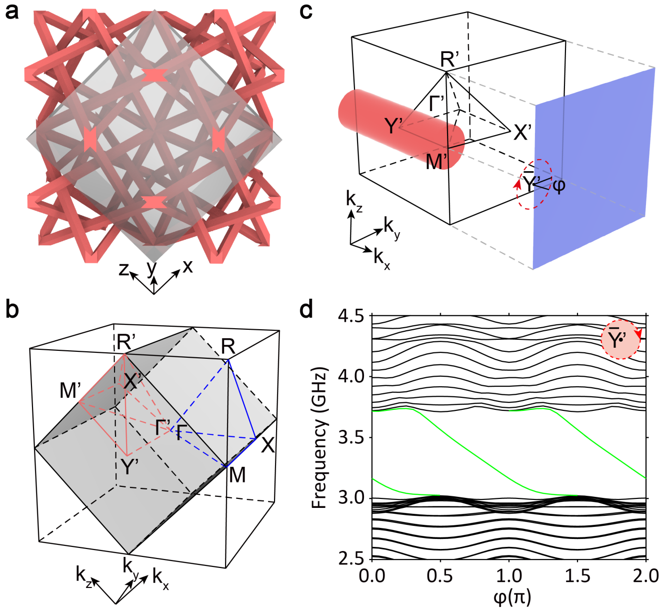

Supplementary Fig. 6 | Numerical evidence for the topological charge of the charge-2 triple point. a A super cell of the 3D metallic-mesh photonic crystal (marked by a grey cuboid), with lattice constant of a = 15 mm along [010] direction, and $\sqrt{2}a$ along [100] and [001] directions. **b** The larger cube (grey cuboid) represents the 3D Brillouin zone of the photonic crystal with unit cell (super cell), where the blue (red) lines display its high-symmetry lines. **c** The 3D Brillouin zone of the super cell and its surface projection on the k_y-k_z plane. The red dashed circle encircling the $\bar{\mathrm{Y}}'$ point is the projection of the red tube oriented along k_x direction. **d** The surface dispersion along the clockwise circular path around the $\bar{\mathrm{Y}}'$ with a radius of 0.23 π/a, at which the charge-2 triple point locates. The green curves represent the topological surface states.

Supplementary Note 10. The evolution of maximally charged WP under $C_{3,111}$ symmetry breaking.

In this section, we give a detailed example of the evolution of maximally charged WP when $C_{3,111}$ symmetry is broken by compressing the structure along [001] direction. Supplementary Fig. 7a shows the unit cell in the tetragonal lattice, with lattice constant along [100], [010] and [001] directions being a, a, and 0.8a (a = 15 mm), respectively. With uniaxial stress along [001] direction, the C_{4x} and C_{4y} symmetries of the system are also broken, while C_{4z}, C_{2x}, $C_{2,110}$, and time-reversal symmetry τ are preserved. The SG of the system changes from No. 207 to No. 89.

We then study the transformation of the WPs under this symmetry breaking. For the previous maximally charged WP at Γ, the corresponding perturbation term in the

leading order can be written as

$$H_{\Gamma,\text{pert}} = \Delta_1 \begin{bmatrix} 1 & 0 \\ 0 & -1 \end{bmatrix}, \tag{43}$$

with Δ_1 being a real parameter. It is easy to check that $H_{\Gamma,\text{pert}}$ commutes with all persevered symmetries C_{4z}, C_{2x}, $C_{2,110}$ and τ, and does not commute with the broken symmetries $C_{3,111}$, C_{4x} and C_{4y}. Then, the modified effective Hamiltonian reads,

$$\begin{aligned} H'_\Gamma &= H_\Gamma + H_{\Gamma,\text{pert}} \\ &= c_1 + c_2 k^2 + \begin{bmatrix} \frac{k_x^2 + k_y^2 - 2k_z^2}{\sqrt{3}} c_3 + \Delta_1 & c_3\left(k_x^2 - k_y^2\right) + i c_4 k_x k_y k_z \\ c_3\left(k_x^2 - k_y^2\right) - i c_4 k_x k_y k_z & -\frac{k_x^2 + k_y^2 - 2k_z^2}{\sqrt{3}} c_3 - \Delta_1 \end{bmatrix}. \end{aligned} \tag{44}$$

When Δ_1 is finite, the maximally charged WP would be gapped and split into two charge-2 WPs or four charge-1 WPs depending on the value of Δ_1. According to our simulated dispersion, as shown in Supplementary Fig. 7b, we find $\frac{\Delta_1}{c_3} < 0$, and the maximally charged WP splits into four charge-1 WPs residing at Γ-M paths with $\mathbf{k} = \left(\pm\sqrt{-\frac{\sqrt{3}\Delta_1}{2c_3}}, \pm\sqrt{-\frac{\sqrt{3}\Delta_1}{2c_3}}, 0\right)$ (red dots in Supplementary Fig. 7b and 7c).

For the previous charge-2 triple point at R, the perturbation term in the leading order can be written as,

$$H_{\text{R,pert}} = \Delta_2 \begin{bmatrix} 0 & 0 & 0 \\ 0 & 0 & 0 \\ 0 & 0 & 1 \end{bmatrix}, \tag{45}$$

with Δ_2 being a real parameter. Then the modified Hamiltonian is

$$H'_\text{R} = c_5 + c_6\, \mathbf{k} \cdot \mathbf{L} + H_{\text{R,pert}} = c_5 + \begin{bmatrix} 0 & -ic_6 k_z & -ic_6 k_y \\ ic_6 k_z & 0 & -ic_6 k_x \\ ic_6 k_y & ic_6 k_x & \Delta_2 \end{bmatrix}. \tag{46}$$

It shows that for finite Δ_2, the charge-2 triple point at R is transformed into two charge-1 WPs locating at M-A paths with $k_z = \pm\frac{\Delta_2}{c_6}$, consistent with the simulation results (see orange dots in Supplementary Fig. 7b and 7c).

Before symmetries breaking, there are three identical M points in the 3D Brillouin zone, which are guaranteed by the $C_{3,111}$ symmetry. Each M point hosts a charge-2 WP. However, the two M points in $k_z = \pi$ plane are no longer equivalent to the M point in $k_z = 0$ plane when the $C_{3,111}$, C_{4x} and C_{4y} symmetries are broken. As a result, the charge-2

WPs in $k_z = \pi$ plane would be gapped, while that in $k_z = 0$ plane remains unchanged. Explicitly, the corresponding perturbation term for the charge-2 WPs in $k_z = \pi$ plane in the leading order can be written as,

$$H_{\mathrm{M,pert}} = \Delta_3 \begin{bmatrix} 1 & 0 \\ 0 & -1 \end{bmatrix}, \tag{47}$$

with Δ_3 being a real parameter. The modified Hamiltonian is,

$$H'_{\mathrm{M}} = c_7 + c_8(k_z^2 + k_x^2) + c_9 k_y^2$$
$$+ \begin{bmatrix} c_{10}(k_z^2 - k_x^2) + \Delta_3 & c_{11}k_z k_x + ic_{12}k_y \\ c_{11}k_z k_x - ic_{12}k_y & -c_{10}(k_z^2 - k_x^2) - \Delta_3 \end{bmatrix}. \tag{48}$$

When Δ_3 is finite, the charge-2 WP in $k_z = \pi$ plane would split into two charge-1 WPs. According to our simulated dispersion, we find $\frac{\Delta_3}{c_{10}} < 0$, and the charge-2 WP becomes two charge-1 WPs residing at R-X paths with $k_z = \pm\sqrt{-\frac{\Delta_3}{c_{10}}}$, as marked by the purple dots in Supplementary Fig. 7b and 7c.

The topological charges of these WPs are also confirmed by the evolution of the Wannier centres on the spheres enclosing the WPs, as shown in Supplementary Fig. 7d. Thus, the total topological charge of the first band is zero, which is consistent with the no-go theorem[13].

Since $C_{3,111}$ symmetry is broken, (001) and (100) ((010)) surfaces are no longer identical. In the following, we numerically calculate the topological surface states on (001) and (100) surfaces. For (001) surface, considering the band projection along k_z direction, the charge-1 WPs at Γ-M paths are projected at $\bar{\Gamma} - \bar{\mathrm{M}}$ paths, and the charge-1 WPs at R-X paths are projected at $\bar{\mathrm{X}}$ and $\bar{\mathrm{Y}}$, while the charge-1 WPs at A-M paths and charge-2 at M coincide, leading to the cancellation of their topological charges. Therefore, there are topological surface states (green curves in Supplementary Fig. 8a) on (001) surface, connecting the projections of the oppositely charged WPs at the path $\bar{\Gamma} - \bar{\mathrm{M}}$ and $\bar{\mathrm{X}}$ or $\bar{\mathrm{Y}}$. Besides, there are four Fermi arcs on the k_x-k_y plane, as schematically and numerically shown in Supplementary Fig. 8b. For the (100) surface, after the band projection along k_x direction, there are WP projections with charge of −2 at $\bar{\Gamma} - \bar{\mathrm{Y}}$ paths, that with charge of +1 at $\bar{\Gamma} - \bar{\mathrm{Z}}$ paths, and that with charge of +2 at $\bar{\mathrm{Y}}$

(see Supplementary Fig. 9a and 9b). As a result, there are still topological surface states and four Fermi arcs connecting the projections of the oppositely charged WPs, regardless of the broken C_{4x} symmetry.

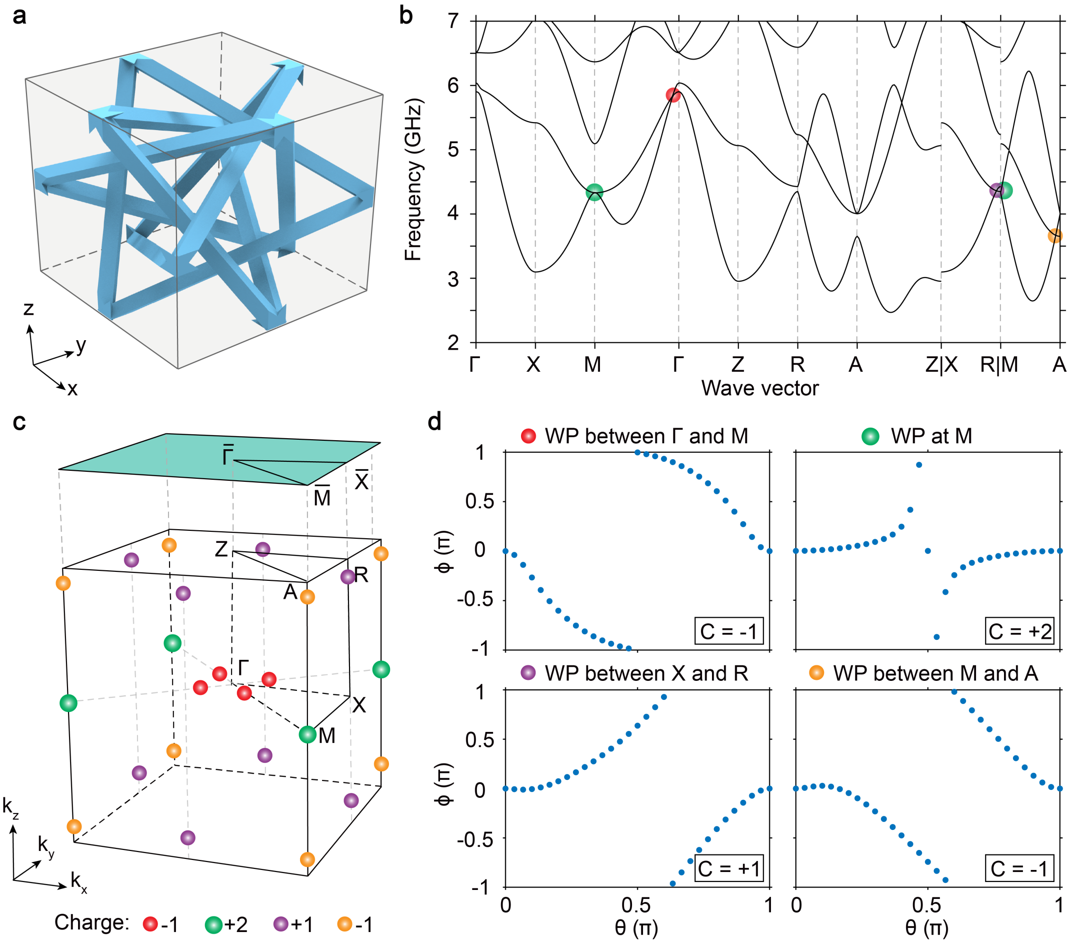

Supplementary Fig. 7 | The evolution of maximally charged WP under $C_{3,111}$ symmetry breaking. **a** A compressed unit cell of the tetragonal lattice. The lattice constant along [100], [010] and [001] directions are a, a, and 0.8a, respectively, with a = 15 mm. **b,c** Band structure (**b**) and 3D first Brillouin zone (**c**) of the 3D photonic crystal, with green (red, purple and orange) dots indicating the charge-2 WPs (charge-1 WPs). **d** Evolution of the Wannier centres for the first band on the spheres enclosing the charge-2 WP and charge-1 WPs, using Wilson loop method.

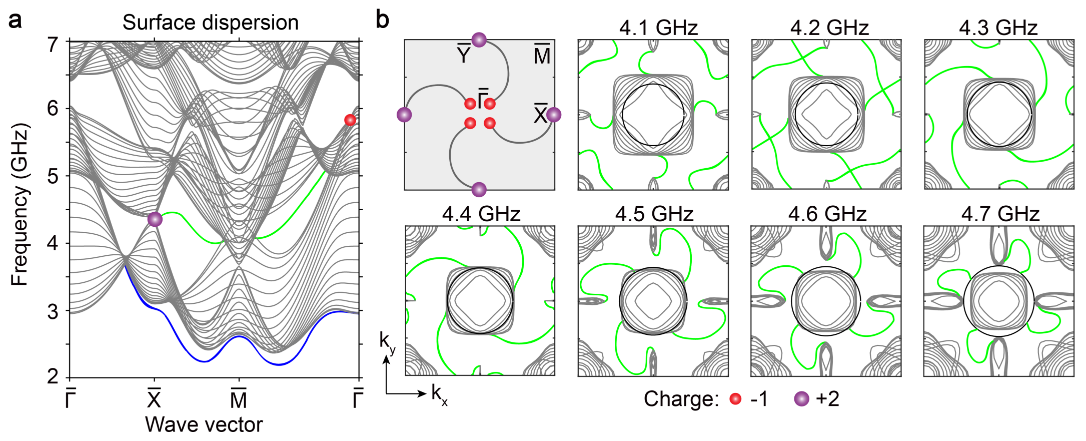

Supplementary Fig. 8 | The topological surface states and Fermi arcs on k_x-k_y plane when $C_{3,111}$ symmetry is broken. **a** The surface dispersion along the high-symmetry line $\bar{\Gamma}-\bar{X}-\bar{M}-\bar{\Gamma}$. Green, blue and grey curves represent the topological surface states, trivial surface state, and bulk states, respectively. Red (purple) dot displays the projection of WPs with topological charge of −1 (+2). **b** Schematic of the four Fermi arcs, and surface iso-frequency contours from 4.1 GHz to 4.7 GHz. Green (grey) curves represent the dispersions of topological surface states (bulk states). Black circles are the light cones.

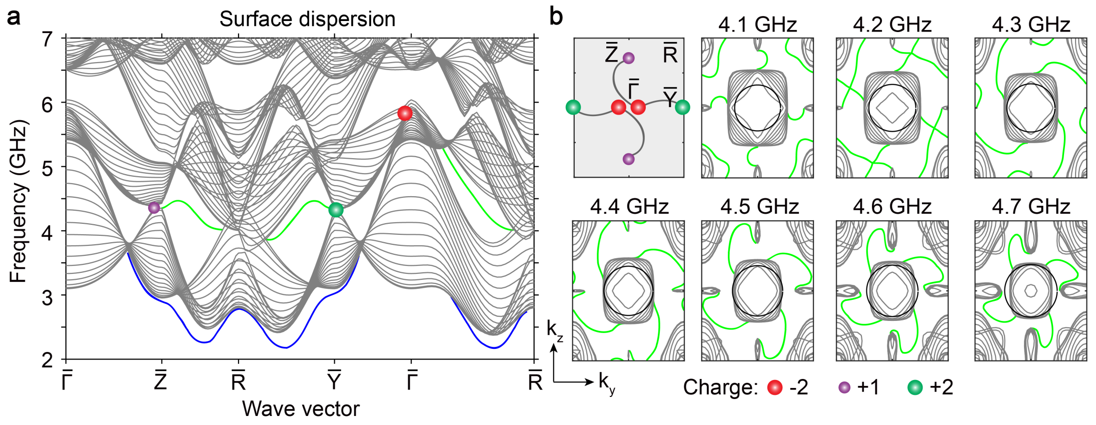

Supplementary Fig. 9 | The topological surface states and Fermi arcs on k_y-k_z plane when $C_{3,111}$ symmetry is broken. **a** The surface dispersion along the high-symmetry line $\bar{\Gamma}-\bar{Z}-\bar{R}-\bar{Y}-\bar{\Gamma}-\bar{R}$. Green, blue and grey curves represent the topological surface states, trivial surface state, and bulk states, respectively. Red, purple and green dots display the projections of WPs with topological charge of −2, +1 and +2, respectively. **b** Schematic of the four Fermi arcs, and surface iso-frequency contours from 4.1 GHz to 4.7 GHz. Green (grey) curves represent the dispersions of topological surface states (bulk states). Black circles are the light cones.

Supplementary Note 11. Design of a 3D acoustic crystal with a maximally charged WP.

In this section, we show the possibility of realising a maximally charged WP in a 3D acoustic crystal by using our symmetry-based design principle. The designed 3D acoustic crystal is shown in Supplementary Fig. 10a, which has a similar structure as the photonic crystal in the main text, expect that the square tubes are filled with air, and the rest are hard materials, such as metals or resin. In this way, the sound propagates along the tubes. The lattice constant of this cubic lattice is $a = 20$ mm, and the width of the square rods is $w = 2.5$ mm.

The band structure is numerically calculated, as shown in Supplementary Fig. 10b, where the second and third bands cross with each other, forming a maximally charged WP at Γ (red dots), a charge-2 triple point at R (blue dots), and several charge-1 WPs at general momenta (green, orange and yellow dots). We then calculate the topological charges of these WPs by utilising the Wilson loop method. Supplementary Fig. 10d displays the evolution of the Wannier centres of the second band on the spheres enclosing these band degenerate points, confirming that the topological charge of the maximally charged WP at Γ is four. Besides, the total topological charge of the second band is zero, which is consistent with the no-go theorem[13].

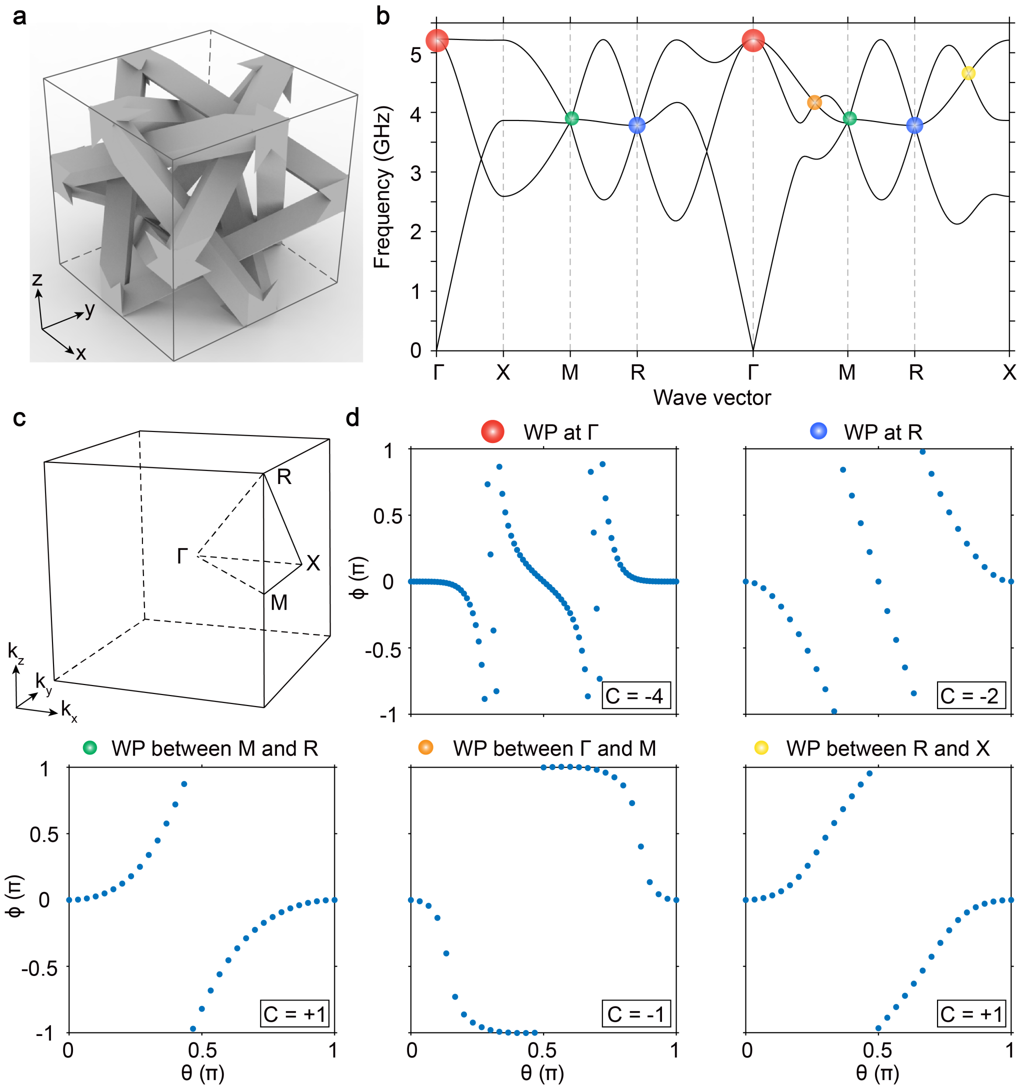

Supplementary Fig. 10 | Design of a 3D acoustic crystal with a maximally charged WP. a A unit cell of the 3D acoustic crystal in the cubic lattice with lattice constant a = 20 mm. The square tubes (the grey regions) are filled with air, and the rest are hard materials. **b,c** Band structure (**b**) and 3D first Brillouin zone (**c**) of the 3D acoustic crystal. The red (blue) dots indicate the maximally charged WP at Γ (charge-2 triple point at R). The green, orange and yellow dots represent the charge-1 WPs at general momenta. **d** Evolution of the Wannier centres for the second band on the spheres enclosing the maximally charged WP, the charge-2 triple point, and the charge-1 WPs, using Wilson loop method.

Supplementary Note 12. The projections of WPs on the lowest three bands on k_x-k_y plane, and the surface dispersion.

In this section, we discuss the projected WPs on the lowest three bands and the corresponding surface states. Supplementary Fig. 11a displays the projected WPs on k_x-k_y plane, where the red dot at $\bar{\Gamma}$ and green dots at $\bar{X}$ involve the first and second

bands, while other dots with dashed outlines involve the second and third bands. Supplementary Fig. 11b plots the corresponding surface dispersion, which clearly indicates the Fermi arcs from the WPs between the second and third bands are separated from that of the maximally charged WP—the former exists roughly above 4.8 GHz, and the latter appears roughly below 4.8 GHz.

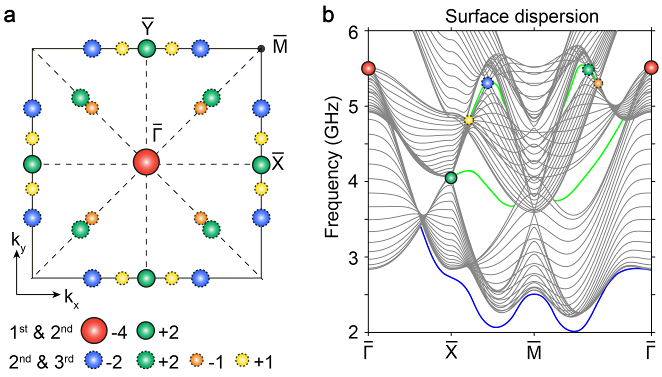

Supplementary Fig. 11 | Projected WPs of the lowest three bands on k_x-k_y plane, and the corresponding surface states. a Projected WPs on k_x-k_y plane. The red and green dots with solid outlines represent the WPs involving the first and second bands, while other dots with dashed outlines indicate the WPs involving the second and third bands. **b** Surface dispersion along the high-symmetry line $\bar{\Gamma}-\bar{\mathrm{X}}-\bar{\mathrm{M}}-\bar{\Gamma}$. Green (blue) curves represent the topological surface states (trivial surface states). Grey curves display the bulk states.

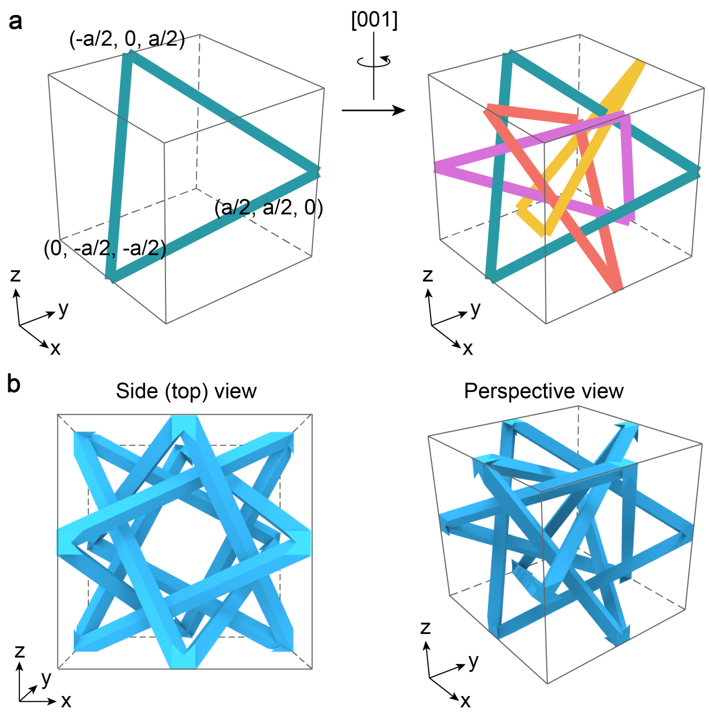

Supplementary Fig. 12 | Detailed schematic of the unit cell of the self-supporting 3D photonic crystal. a A unit cell constructed by rotating a triangle along [001] direction for 90, 180, and 270 degrees. Left panel: a triangle with vertices locating at (−a/2, 0, a/2), (a/2, a/2, 0) and (0, −a/2, −a/2). Right panel: a unit cell after three rotations, which consists of four triangles. Therefore, our photonic crystal has C_3 rotation symmetry along [111] direction, and C_4 rotation symmetry along [100], [010], and [001] directions. **b** Side/top view, and perspective view of the unit cell.

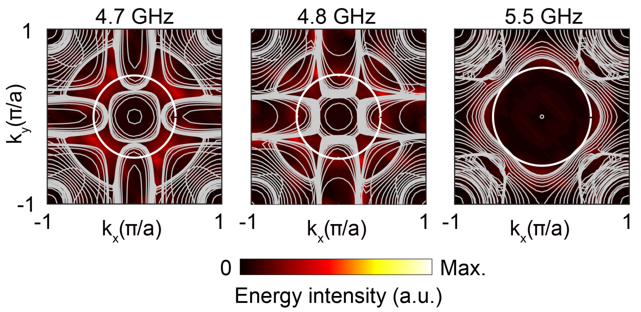

Supplementary Fig. 13 | Measured surface iso-frequency contours at 4.7, 4.8 and 5.5 GHz. Green (grey) curves represent the dispersions of topological surface states (bulk states). White bold circles are the light cones. The colourmap measures the energy density. The plotted range for the iso-frequency contours is $[-\pi/a, \pi/a]^2$.

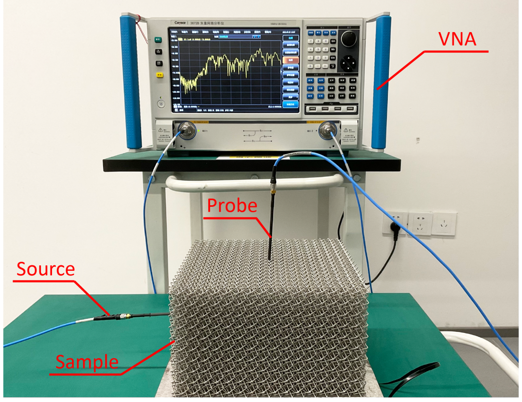

Supplementary Fig. 14 | Experimental setup consisting of a vector network analyser (VNA), two cables, a source, a probe, and a fabricated sample.

Supplementary references:

1. Zhang, T., Takahashi, R., Fang, C. & Murakami, S. Twofold quadruple Weyl nodes in chiral cubic crystals. *Phys. Rev. B* **102**, 125148 (2020).
2. Cui, C., Li, X.-P., Ma, D.-S., Yu, Z.-M. & Yao, Y. Charge-four Weyl point: Minimum lattice model and chirality-dependent properties. *Phys. Rev. B* **104**, 075115 (2021).
3. Bradlyn, B. *et al.* Beyond Dirac and Weyl fermions: Unconventional quasiparticles in conventional crystals. *Science* **353**, aaf5037 (2016).
4. Tang, P., Zhou, Q. & Zhang, S.-C. Multiple types of topological fermions in transition metal silicides. *Phys. Rev. Lett.* **119**, 206402 (2017).
5. Zhang, T. *et al.* Double-Weyl phonons in transition-metal monosilicides. *Phys. Rev. Lett.* **120**, 016401 (2018).
6. Fang, C., Lu, L., Liu, J. & Fu, L. Topological semimetals with helicoid surface states. *Nat. Phys.* **12**, 936–941 (2016).
7. Yu, Z.-M. *et al.* Encyclopedia of emergent particles in three-dimensional crystals. *Sci. Bull.* **67**, 375-380 (2022).
8. Wang, H.-X., Guo, G.-Y. and Jiang, J.-H. Band topology in classical waves: Wilson-loop approach to topological numbers and fragile topology. *New J. Phys.* **21**, 093029 (2019).
9. Yang, Y. *et al.* Topological triply degenerate point with double Fermi arcs. *Nat.*

Phys. **15**, 645-649 (2019).

10. Sanchez, D. S. *et al.* Topological chiral crystals with helicoid-arc quantum states. *Nature* **567**, 500-505 (2019).
11. Rao, Z. *et al.* Observation of unconventional chiral fermions with long Fermi arcs in CoSi. *Nature* **567**, 496-499 (2019).
12. He, H. *et al.* Observation of quadratic Weyl points and double-helicoid arcs. *Nat. Commun.* **11**, 1820 (2020).
13. Nielsen, H. B., Ninomiya, M. A no-go theorem for regularizing chiral fermions. *Phys. Lett. B* **105B**, 219-223 (1981).
